# Operations for Fissure of the Soft and Hard Palate

**Published:** 1843-04-29

**Authors:** 


*Operations for Fissure of the Soft and Hard Palate. (Palatoplastie.)* By J. Mason Warren, M. D, Boston, 1843. 8vo. pp. 11.

The present publication is designed to show the successful application of a modification of Professor Roux's operation for fissure of the palate. Dr. Warren thus describes the operation he has adopted:

"The patient is placed on a low seat, in a strong light, his head firmly supported on the breast of an assistant, who raises or depresses it as circumstances may require. He is directed to keep the jaws widely separated, to retain any blood which may collect as long as possible, so as not to embarrass the operator, and restrain all efforts at coughing. To do this will require constant warnings and encouragement on the part of the surgeon, as there is a natural tendency to close the mouth as soon as any pain is felt, or there arises any collection of blood or mucus in the fauces which interferes with respiration. The use of a speculum, as directed by some operators, is altogether inadmissible; it not only obscures the light, but also prevents the proper manœuvres of the instruments. The mucous membrane of the hard palate is now to be carefully separated from the bones with a long, double-edged bistoury, curved on its flat side, and is rather pealed than dissected off, from the difficulty of making any sawing motion with the knife in this confined situation, the obstacles always being greater in proportion to the obliquity of the palatine vault. As the dissection approaches to the connection of the soft parts with the edges of the ossa palati, where the muscles are attached and the union most intimate, great care must be taken or the mucous membrane will be perforated, and from these causes I have found this part of the operation to be the most embarrassing. As soon as this dissection is terminated, it will generally be found that by seizing the soft palate with a forceps it can be easily brought to a median line. If the fissure is wide, and this cannot be effected, I have found the following course to be invariably followed by success. The soft parts being forcibly stretched, a pair of long, powerful French scissors, curved on the flat side, are carried behind the anterior pillars of the palate; its attachments to the tonsil and to the posterior pillar are now to be carefully cut away, on which the anterior soft parts will at once be found to expand, and an ample flap be provided for all desirable purposes.

The edges of the palate may now be made into a raw surface by seizing them on either side with a hooked forceps and removing a slip with the scissors or a sharp-pointed bistoury. Our next object is to insert the ligatures, and for this purpose an immense armory of instruments have been invented. After the trial of nearly all of them I have found the most simple to be the most effectual. A small curved needle being armed with a strong silk thread, confined in a forceps with a movable slide, is introduced to the upper edge of the fissure, the needle being carried from before backwards on the left side, and from behind forwards on the right, or vice versa. In this manner, three or four more ligatures may be successively introduced. The patient is now requested to clear his throat of mucus and blood, the ligatures are wiped dry and waxed, and tied with deliberation, beginning at the upper, and proceeding gradually downwards, waiting a little between each ligature, in order to allow the throat to accommodate itself to this sudden and almost insupportable tension of the soft parts. No forceps are required for holding the first knot while the second is tied; the object is better effected by using the surgeon's knot; that is, by making two turns of the thread instead of one, and by enjoining perfect quiet on the patient for the moment, until the second knot is tied. It has been advised by some surgeons to wait a certain length of time, after the cutting part of the operation, before inserting the ligatures, five or six hours for instance, to allow all bleeding from the wound to cease. This appears to me a useless prolonging of the patient's suffering, and entirely unnecessary. I have never seen, in a single instance, either in the operations of surgeons abroad or in my own experience, any hemorrhage that a little iced water, or the pressure for a short period with the finger, would not easily arrest. The after treatment will not here require any notice, as it has been sufficiently noted in the previous detail of the cases.

In all the operations of this kind which I have lately had occasion to perform, the ligatures have been removed at the end of forty-eight hours, or at the farthest three days, and to this circumstance may be partly attributed the successful termination. If the threads be allowed to remain until the 4th, 5th, or 6th day, as recommended and practised by Roux, the apertures left by them will be of such magnitude as almost to approach each other, and to weaken the parts so as to cause a separation on any untoward motion of the patient."

M. Roux has lately published a resumé of his cases. Where there is simple fissure of the palate, two out of three cases succeed. Where there is complication with fissure of the hard palate, only one out of three is successful. In these cases he still cuts away the soft palate transversely from the palatine bones, and stretches the flaps across the chasm, thus leaving an operation in the bones to be artificially cured. Dr. Warren has obtained by his method the extraordinary success of 13 out of 14. In the unsuccessful case there was so great deficiency of the soft parts that Dr. W. stated to the patient the possibility of a failure.

---

The twenty-sixth "Annual Report on the state of the Asylum for the Relief of Persons deprived of their Reason," located at Frankford, Pennsylvania, is before us. It shows the Institution to be in a prosperous condition, and the plan of treatment pursued to be similar to the one now universally adopted.

